# First report of the cystic fibrosis transmembrane conductance regulator mutation c.1521_1523delCTT (p. Phe508del) in two Qatari patients with cystic fibrosis

**DOI:** 10.5339/qmj.2021.24

**Published:** 2021-07-26

**Authors:** Atqah AbdulWahab, Amal AlNaimi, Basel Habra, Ibrahim Janahi

**Affiliations:** ^1^Department of Paediatric Pulmonology, Sidra Medicine, Doha, Qatar E-mail: atqah2015@gmail.com; ^2^Department of Paediatric Medicine, Hamad Medical Corporation, Doha, Qatar; ^3^Department of General Paediatric, Weill Cornell Medicine-Qatar

**Keywords:** cystic fibrosis, p. Phe508del mutation, Qatar

## Abstract

We report two cases of Qatari children with cystic fibrosis (CF) from different families presenting the homozygous CFTR 1521_1523delCTT (p. Phe508del) mutation with classic CF phenotypes. This gene mutation is considered the second CF mutation identified in Qatar. Herein, we review the frequency and distribution of this mutation in Arab countries.

## Introduction

Cystic fibrosis (CF) is a monogenic autosomal recessive disorder that affects multiple organ systems of the body. It is characterized by a highly variable clinical presentation, which involves the pulmonary, digestive, sweat gland, and reproductive systems.^1,2^ CF is caused by mutations in CFTR (cystic fibrosis transmembrane conductance Regulator) gene and >2,000 mutations of CFTR gene have been identified.^3^ Furthermore, depending on the fate of the protein encoded by CFTR gene, six different classes of variants have been identified.^2^ The most common mutation in CFTR gene worldwide is c.1521_1523delCTT (p. Phe508del = F508del), which deletes a three-base-pair (in-frame) at position 508 in the coding region of the gene, leading to loss of a phenylalanine residue (c.1521_1523delCTT). Thus, this leads to production of a misfolded variant of the protein, which is quickly degraded. This mutation was identified in the process of the initial sequencing of CFTR gene.^4^

The frequency of p. Phe508del varies by geographic region. It occurs in 86.4% of US patients (46.5% are homozygous for the mutation, with ∼40% carrying at least one allele) and the frequency in Europe varies from a maximum of 100% in the isolated Faroe Islands of Denmark, to a minimum of 20% in Turkey.^5–7^

Earlier in 2019, Al-Sadeq et al.,^8^ in a systematic review, reported a spectrum of CF mutations in 22 Arab countries and the CFTR mutation c.1521_1523delCTT (p.Phe508del) was not found in Qatar. For the first time, we report this type of CFTR mutation in two Qatari patients belonging to different Arab tribes. This mutation is considered the second CFTR mutation identified among Qatari CF patients where the most common CFTR mutation that was reported previously in Qatari CF patients is the homozygous CFTR mutation c.3700 A>G (p. Ile1234Val = I1234V).^9^

## Case presentation

Case 1: This is a 3-year-old Qatari girl presented with recurrent wheeze, lower respiratory infections, frequent stool (4–6 times per day [greasy and offensive odor]), and failure to thrive since 3 months of age. The Qatari parents are first cousins with a negative family history of CF. CF was diagnosed at the age of 9 months based on classic CF phenotypic presentation and was confirmed by repeated elevated sweat chloride level of 92 mmol/L & 95 mmol/L, respectively. The sweat test was performed with Macro duct collection system (Wescor, INC-USA) and advanced sweat chloride analyzer. Her CFTR gene mutation revealed homozygous c.1521_1523delCTT (p. Phe508del). Stool elastase was < 100 μgE/g, indicating exocrine pancreatic insufficiency.^10^ Broncho alveolar lavage culture grew *Pseudomonas aeruginosa* at the age of 9 months,where she received two weeks of intravenous cefepime and amikacin and airway clearance therapy, which resulted in marked improvement. Her chronic diarrhea also improved on pancreatic enzyme replacement therapy, which was started at the time of diagnosis. Both weight and height were maintained between the 25th and 50th centiles and she is presently in a stable clinical condition.

Case 2: This is a 10-year-old boy who is the third child of a Qatari first-generation cousins (from a different tribe than case 1). He was presented with a history of chronic diarrhea and inadequate weight gain since the age of 2 years. CF was diagnosed at the age of 5 years, where he had repeated elevated sweat chloride concentration of >100 mmol/l, stool elastase of < 100 μgE/g, which indicated exocrine pancreatic insufficiency, and a CFTR gene mutation of homozygous c.1521_1523delCTT (p. Phe508del). His first episode of chest infection was At the age of 8 years. His sputum culture grew *Pseudomonas aeruginosa*, and he received nebulized tobramycin and airway clearance therapy for a period of 1 month. Subsequently, improvement was observed, and he is presently maintaining an appropriate growth pattern and lung function.

## Discussion

The incidence of CF in the Middle East varies according to ethnic background and the degree of consanguinity.^11^ Estimates were in the range of 1 in 2,560 to 1 in 15,876 and the prevalence of CF in the Middle East was reported to be 1 in 2500–5000.^12^

In Qatar, a total of 45 children were diagnosed with CF, of which 32 (71.1%) were Qatari. In the present case study, nine different CFTR mutations were identified (Table 1), with majority (n = 30; Qatari CF children; 66.67%) having homozygous c.3700 A>G (p. Ile1234Val), which has been recently reported.^13^ The second common mutation was homozygous p. Phe508del, which was identified in 7 (15.56%) CF children, including 2 Qatari of the present cases, 1 Syrian, 2 Pakistani, and 2 Bangladeshi. Other CFTR mutations are listed in Table 1.

It has been reported that there are specific CFTR mutations that appear to be more common throughout the Middle East, but are rarely observed elsewhere. In some cases, certain CFTR gene mutations may be specific to a subset of the people in the Middle East who are defined by a common ethnic background. For instance, the mutation CFTR c.3700 A>G (p.Ile1234Val) in certain Arab tribes, which was reported previously as the only CFTR gene mutation identified in Qatar, was associated with a high frequency of consanguinity.^9^

Although p. Phe508del gene mutation is more frequently found in Europe than in the Middle East, it is relatively common in some Arab countries (Table 2). A study conducted in Lebanon by Farra et al.^14^ revealed that p. Phe508del mutation was the most common CF mutation, with a frequency rate of 34%. In Jordan, the reported frequency rate was 41.5% and, in Syria, it was 18%.^15,16^ In Egypt, analysis of p.Phe508del homozygous mutation in CF patients was 22.2%.^17^ This mutation is reportedly the most frequent CFTR mutation in Algeria (18.75%) and Tunisia (56%).^18,19^ In Saudi Arabia, this mutation constitutes 11% of CFTR mutations in CF patients and, in Bahrain, this mutation was found in < 8% of CF patients.^20,21^

The frequency of distribution further changes in Oman and United Arab Emirates, where p. Phe508del mutation accounts for 95% of affected families with CF; however, it was exclusively found in patients of Baluchi descent.^22–24^ This is similar to the findings regarding the high frequency of p. Phe508del in non European CF patients, in which the mutation was found in approximately 60% (Pakistani) and 100% (Pakistani Baluch) of patients with CF.^24^

The Baluch ethnic group resides in Iran, Baluch area of Pakistan (Baluchistan), and Afghanistan. Historically, these people have traveled in successive waves to Punjab, India, and the Arabian Gulf countries (Oman and the UAE) and probably from there to the first wave of emigrants who brought the mutation to Europe.^24^ It was proposed that p. Phe508del CF mutation probably originated from Baluchistan.^25^

Herein, we report the first two cases of CF with this mutation among Qatari families who are not from a Baluchi descendant, but from different tribes that are usually associated with severe and typical CF phenotypes than the most common CFTR mutation in Qatar (p. Ile1234Val with mild CF).

## Conclusion

Our reported cases further underline the importance for clinicians to consider different types of CFTR mutations in patients who present classic CF phenotype and to broaden the genetic mutational analysis consideration of CFTR among Qatari patients with CF.

## Figures and Tables

**Table 1:** Spectrum of CFTR mutations in Qatar

Traditional (Legacy name)	Nucleotide (DNA level)	Amino Acid (Protein level)	Total CF pts = 45 (CF children)	Nationality	Percentage %

Homozygous I1234V mutation	c.3700 A>G	p.Ile1234Val	30	Qatari	66.7

Homozygous fig508del	c.1521_1523delCTT	p.Phe508del	7	2 Qatari 1 Syrian 2 Bangladeshi 2 Pakistani	15.6

Homozygous Y569D	c.1705T- > G	p.Tyr569Asp	2	2 Pakistani	4.4

Homozygous 3120+1G>A	c.2988+1G>A (splice donor)	Intronic	1	1 Pakistani	2.2

Homozygous Deletion40 KB Exon4-9			1	Iranian	2.2

Compound heterozygous	c.2805-2810 delins TCAGA, c.2290C>T	p.Leu941Ter p.Arg764Ter	1	Bangladeshi	2.2

Homozygous	c.2997-3000delAATT	p.Ile1000	1	Egyptian	2.2

Homozygous 2043delG	c.1911delG	p.Gln637HisfsX26	1	Syrian	2.2

Compound heterozygous G85E,R1066C	c.254G>A c.3196C>T	p.Gly85Glu p.Arg1066Cys	1	Jordan	2.2


**Table 2:** Number of CF patients with the CFTR mutation c.1521_1523delCTT (p. Phe508del) in Arab countries CF: cystic fibrosis, n: number

Country	CF Patients (n)	Year publication	Reference number

Saudi Arabia	46	2020	19

United Arab Emirates	8	1997	21

Oman	3	2000	22

Bahrain	2	2002	20

Lebanon	23	2010	13

Jordan	32	2019	14

Syria	5	2018	15

Egypt	22	2014	16

Tunisia	19	2018	17

Alegria	4	2008	18

Qatar	2	2021	Present paper

